# Lithotripsy

**Published:** 1838-01-31

**Authors:** 


### DOMESTIC SUMMARY.

### LITHOTRIPSY.

Dr. Randolph performed this operation a few days since, in the Pennsylvania Hospital, upon a female child, four years old, with Heurteloup's instrument of the largest size. The stone was caught twice, and crushed without any difficulty, and the child has passed several fragments since. No bad symptoms have occurred nor has the girl been at all confined to her bed. [Particulars in a future number.]

---

Adlard and Saunders of New York have in press, Dr. A. H. Stevens' two Lectures on *Lithotomy*, and one on the *Diseases of the Joints.* The former will be illustrated by Plates showing a new mode of operation with the author's *Prostatic Bisector.*

The same publishers also announce as in preparation, *A Treatise on Midwifery, Practically Considered, by* Gunning S. Bedford, M. D., *Lecturer on Midwifery and the Diseases of Women and Children, in the city of New York.* This work will consist of about 400 pp. 8vo. and will be accompanied by Plates, illustrative of the important features of Obstetric Science.

